# The impact of tele-stewardship on rural and suburban pediatric ambulatory antibiotic prescribing

**DOI:** 10.1017/ash.2026.10353

**Published:** 2026-04-21

**Authors:** Matthew James Peworchik, Ritu Banerjee, Henry Domenico, Sophie E. Katz

**Affiliations:** 1 Pediatrics, Vanderbilt University Medical Center, USA; 2 Vanderbilt University Medical Center, USA

## Abstract

**Objective:**

Developing, implementing, and evaluating the effectiveness of outpatient pediatric antimicrobial stewardship interventions via tele-stewardship

**Design:**

Baseline data collected between January and December 2022. Intervention data collected from February 2023 to December 2024. Interrupted time series with regression discontinuity analysis used to compare rates of antibiotic prescription between the periods.

**Setting:**

Three pediatric primary care clinics and three emergency departments associated with Vanderbilt University Medical Center that served rural and suburban communities.

**Participants:**

All encounters with patients less than 18 years of age at participating sites.

**Interventions:**

Intervention bundle included patient/caregiver educational materials, antibiotic use commitment posters, prescriber education through quarterly teaching sessions on common pediatric infections, communication skills training, app-based microlearning modules, access to local guidelines using the Firstline app, and quarterly audit and feedback with peer comparison on guideline-concordant antibiotic use.

**Results:**

Among a total of 147,357 encounters (43,157 baseline, 100,200 intervention), overall percent of encounters with one or more antibiotics prescribed decreased from 12.4% to 11.9% (*P* = .01). Percent change varied by site and patient demographics. Overall guideline-concordant prescribing increased significantly for acute otitis media (77.7% baseline vs 85.7% intervention, *P* < .001), streptococcal pharyngitis (73.8% baseline vs 81.7% intervention, *P* < .001), and urinary tract infections (41.9% baseline vs 57.1% intervention *P* < .001). Five-day antibiotic courses increased significantly (6.3% baseline vs 19.7% intervention, *P* < .001). There was a significant decrease in rapid streptococcal testing (10.9% baseline vs 7.6% intervention, *P* < .001).

**Conclusions and Relevance:**

Tele-stewardship interventions were effective in outpatient pediatric primary care and emergency department settings, but effectiveness varied by site.

## Introduction

The negative effects of inappropriate antimicrobial prescribing include adverse events, increased healthcare costs, antibiotic resistance, microbiome disruption, and *C. difficile* infections. Antimicrobial stewardship programs aim to decrease the unnecessary and inappropriate use of antimicrobials. These programs are commonly implemented in inpatient settings, but there is substantial need for such programs in outpatient settings since most antibiotics in the United States are prescribed for outpatients, and as many as 50% of outpatient antibiotic prescriptions may be inappropriate or unnecessary.^1,2^ Inappropriate outpatient antibiotic prescriptions are most common in rural settings, making these locations prime candidates for targeted outpatient antimicrobial stewardship.^3^

Tele-stewardship involves implementing antimicrobial stewardship interventions via telecommunication and information technology. As tele-stewardship does not require on-site stewardship personnel, it may be a practical approach in the wide geographical distribution of rural outpatient medicine. A variety of studies have shown the effectiveness of tele-stewardship^4,5^ although most have focused on implementation in the adult inpatient context. There has been increasing research demonstrating the feasibility and impact of antimicrobial stewardship in outpatient pediatrics,^6^ but no studies have evaluated this in rural settings. In this study, we evaluate the effectiveness of a bundled tele-stewardship intervention implemented in rural and suburban emergency departments and pediatric primary care clinics.

## Methods

### Participating practices

**Figure 1.**
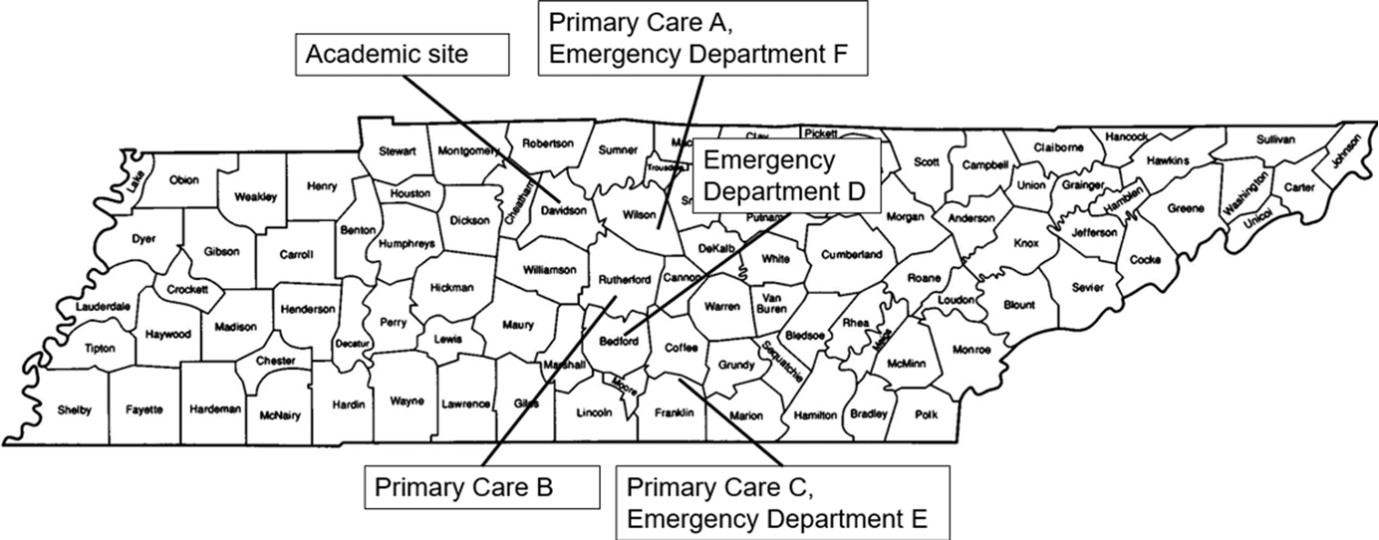
Project sites. Figure illustrates the counties in which the primary care clinics and emergency departments involved in the study were located throughout the state of Tennessee. Home academic site from which the tele-stewardship interventions were carried out is marked as well.

Three pediatric primary care clinics (A, B, and C) and three emergency departments (D, E, and F) were included in the study (Figure 1). These sites consisted of all the non-main campus primary care practices and emergency departments affiliated with Vanderbilt University Medical Center at the time of the study start. The primary care clinics were each staffed by 4–6 prescribers, all but 2 of which were physicians and averaged between 600 and 1,236 encounters per month in the baseline period. The emergency departments were staffed by a rotating team of adult emergency medicine physicians and advanced practice providers (APPs) and averaged between 339 and 401 pediatric encounters per month in the baseline period. The project sites are connected to a central electronic health record (EHR, Epic, Verona, WI) (Figure 1). A Tableau dashboard used for routine antimicrobial stewardship monitoring tracks EHR data at both the clinic and prescriber level and was used to track antibiotic prescriptions, indications, and use of diagnostic tests. No prior stewardship educational efforts had taken place at these sites.

### Study period and outcomes

We evaluated all encounters with patients less than 18 years of age at participating sites.

**Figure 2.**
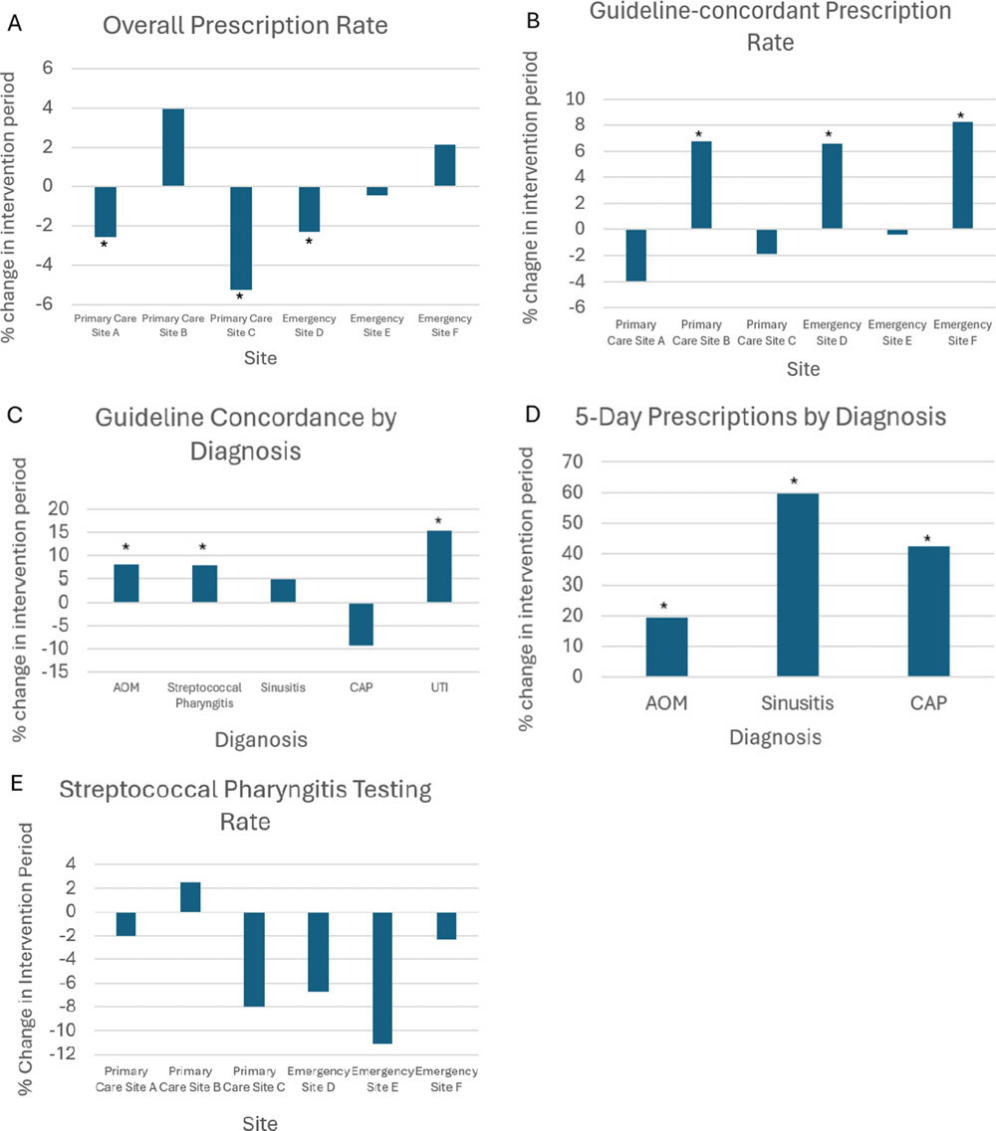
Percent change in outcome. Panel A shows percent change in overall prescription rate by site, with sites that showed a significant improvement marked with an asterisk. Panel B shows percent change in guideline concordant antibiotic choice by site. Panel C shows percent change in guideline concordant antibiotic choice by antibiotic indication. Guideline concordant antibiotics were defined as follows: amoxicillin, penicillin, or amoxicillin/clavulanate for acute otitis media (AOM) or sinusitis, amoxicillin or penicillin for streptococcal pharyngitis and community acquired pneumonia (CAP), and nitrofurantoin or cephalexin for urinary tract infection (UTI). Panel D shows percent change in guideline concordant antibiotic prescription duration by antibiotic indication. Panel E shows percent change in streptococcal pharyngitis testing rates by site. *Denotes statistically significant improvement.

The baseline period was January 2022–December 2022, and the intervention period was February 2023–December 2024, with a washout period in January 2023 that was not included in the analysis. Antibiotic indications were extracted from a required question field at the time of antibiotic order entry which went live in May 2022, and we previously demonstrated highly concordance with documentation in the patient’s chart.^7^ We used ICD-10 codes associated with the order encounter in which an antibiotic was prescribed to extract indication for baseline data prior to May 2022. Concordance with diagnosis-specific national (e.g. acute otitis media (AOM),^8^ streptococcal pharyngitis,^9^ community-acquired pneumonia (CAP),^10^ sinusitis,^11^) and institutional (e.g. urinary tract infection (UTI)) guidelines was assessed for each antibiotic prescription. Concordant antibiotic choice and duration were evaluated separately, as defined in Figure 2. The study’s primary outcome was the percent of all encounters that resulted in an antibiotic prescription. Secondary outcomes included the percent of encounters resulting in a guideline-concordant antibiotic choice for AOM, streptococcal pharyngitis, CAP, sinusitis, and UTI; percent of encounters resulting in a guideline-concordant antibiotic duration for AOM, sinusitis, and CAP; percent of prescriptions with a 5-, 7- or 10-day duration (excluding azithromycin prescriptions); and percent of encounters resulting in a rapid streptococcal pharyngitis antigen or polymerase chain reaction test. Percent of encounters with a return visit anywhere within the healthcare system in the two weeks following the initial encounter was tracked over the course of the study as a balancing measure.

### Interventions

**Figure 3.**
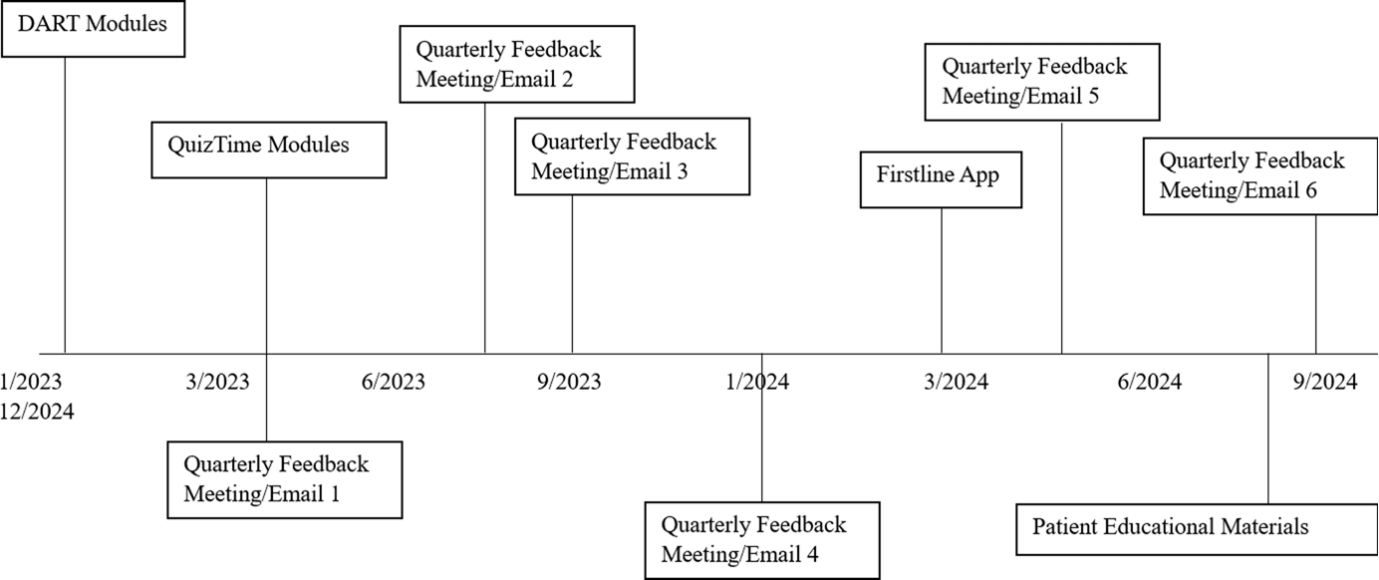
Implementation timeline for various interventions. Feedback meetings and emails occurred quarterly, with the exact time depending on the availability of providers at each site.

The interventions in the tele-stewardship bundle consisted of disseminating patient education materials to participating sites including posters and handouts available in English, Spanish, and Arabic, providing access to educational materials for clinicians including communication skills training modules,^12^ vaccine educational material from the American Academy of Pediatrics (AAP),^13^ and online microlearning modules addressing common causes of inappropriate outpatient antimicrobial prescribing,^14^ creating and disseminating local guidelines for common pediatric outpatient infections based on national guidelines when available, providing access to local clinical guidelines via the Firstline phone app,^15^ and quarterly audit and feedback with peer comparison. The quarterly feedback included an online meeting with each site where overall prescribing trends were reviewed at the clinic site level and de-identified individual prescriber level. Each meeting concluded with a brief teaching point about a common pediatric infection. Each individual prescriber also received a quarterly personalized email that included their rate of antibiotic prescription and rapid streptococcal pharyngitis testing compared to all other prescribers in the study (eFigure). Dates of implementation for each intervention can be found in Figure 3. Continuing medical education credit (CME) credit and Maintenance of Certification (MOC) Part IV Credit were offered for participating in the meetings, review of educational materials and review of feedback reports. The study was approved by the Vanderbilt University Institutional Review Board.

### Statistical analysis

Patient characteristics were summarized using median and interquartile range (IQR) for continuous variables and frequency with percentage for categorical variables. Unadjusted comparisons of outcomes between time periods were performed using Pearson’s χ^2^ test for categorical variables and Kruskall-Wallis test for continuous variables. An interrupted time series with regression discontinuity analysis was used to compare the rates of antimicrobial prescription in the baseline period to the intervention period.^16^ A similar model was used to investigate the secondary outcomes. A mixed-effects logistic regression model was used to estimate the effect of implementing the intervention. All analyses were performed using *R* version 4.4.0 software (*R* Foundation for Statistical Computing, Vienna, Austria).

## Results

Among 147,357 total encounters (29.3% baseline, 68% intervention), 106,651 (72.4%) occurred in a pediatric primary care clinic while 40,739 (27.6%) occurred in an emergency department. The median patient age was 5 years (interquartile range 1–10 years). Most patients identified as White (63.0%), non-Hispanic/Latino (70.0%), and had public insurance (65.0%). Additional demographics are shown in Table 1.

**Table 1.** Patient and participating practice characteristics

		Primary care A		Primary care B		Primary care C		Emergency department D		Emergency department E		Emergency department F	
	Overall (N = 147,390)	Baseline (N = 8,016)	Implementation (N = 21,110)	Baseline (N = 14,835)	Implementation (N = 33,184)	Baseline (N = 7,202)	Implementation (N = 19,302)	Baseline (N = 4,069)	Implementation (N = 8,629)	Baseline (N = 4,812)	Implementation (N = 9,417)	Baseline (N = 4,223)	Implementation (N = 8,558)
Age (yrs.)													
Median	5	2	2	4	5	4	5	8	8	6	7	8	8
Sex													
Female (%)	48.30	49.20	46.60	48.8	48.80	46.10	47.60	48.60	48.30	49.90	50.10	49.10	48.60
Race													
Black (%)	14.40	12.80	11.50	22.40	20.20	8.70	8.40	13.00	12.40	10.10	8.10	15.20	17.20
Other (%)	16.80	11.50	15.40	26.90	31.90	3.80	7.10	8.90	14.70	5.00	7.80	5.20	10.70
Prefer not to answer (%)	4.90	3.80	4.70	8.70	10.00	1.10	3.10	0.70	1.20	0.40	0.90	1.30	1.90
White (%)	63.00	68.80	68.20	40.30	37.80	84.70	81.40	73.70	71.50	80.80	83.00	73.60	69.90
Ethnicity													
Hispanic/Latino (%)	16.10	14.10	15.70	21.60	21.60	7.10	11.20	18.90	19.50	10.90	10.20	13.10	13.20
Non-Hispanic/Latino (%)	70.00	70.80	76.40	55.90	59.90	76.40	79.90	60.70	77.30	59.20	84.80	52.40	82.10
Unknown (%)	10.50	7.90	7.60	19.00	18.40	9.10	8.50	4.80	2.80	5.70	4.20	4.80	3.60
Preferred language													
English (%)	82.80	91.20	89.10	65.30	62.30	98.30	95.40	90.50	91.00	94.80	94.90	91.80	90.50
Non-English (%)	17.10	8.80	10.80	34.60	37.60	1.70	4.60	9.40	8.90	5.00	5.00	7.90	9.30
Insurance													
Unknown (%)	9.60	13.20	9.10	14.70	14.20	11.40	14.90	0.80	0.20	1.00	0.10	0.40	0.20
Private (%)	23.40	28.40	31.90	19.90	19.40	25.10	23.10	17.60	20.90	18.70	22.50	26.50	26.30
Public (%)	65.00	57.50	58.30	65.00	65.60	62.80	61.00	77.00	73.20	75.70	72.00	68.50	66.90
Self-Pay (%)	2.00	0.90	0.70	0.50	0.70	0.70	1.10	4.60	5.70	4.50	5.30	4.60	6.60
Provider type													
NP (%)	10.00	0.20	0.00	0	0.00	48.70	39.70	13.90	4.60	9.30	4.00	17.20	6.80
Other (%)	0.70	0.60	0.60	0.30	0.30	0.20	0.10	0.40	3.70	1.90	1.30	0.20	1.00
PA (%)	7.40	0	0	0	0	0	0	15.00	25.50	27.90	27.50	21.50	34.60
Physician(%)	79.80	99.10	99.40	97.80	98.90	49.60	60.10	66.20	59.80	48.40	59.60	58.30	56.10

Overall antibiotic prescription rate decreased slightly from 12.4% (5338/43157) of encounters in the baseline period to 11.9% (11914/100200) of encounters in the intervention period (*P* = .01). Adjusted comparison via regression discontinuity analysis showed an odds ratio of 0.81 (*P* < .001) implying lower odds of antibiotic prescribing in the intervention period. Change in prescription rates varied by individual site (Figure 2). Two primary care clinics showed significant decreases in antimicrobial prescribing, while one showed a significant increase. There was greater variability among the emergency departments, with one having a significant decrease, one having a non-significant decrease, and one having a significant increase in overall prescription rate (Figure 2). There were statistically significant but non-clinically significant changes in prescription rate by race and primary spoken language (eTable).

Overall guideline-concordant prescribing for all indications increased from 44.3% in the baseline period to 47.8% in the intervention period (*P* < .001). Adjusted comparison via regression discontinuity analysis after adjusting for viral season and preexisting time trend showed an odds ratio of 0.78 (*P* = .02) suggesting lower guideline-concordant prescribing in the intervention period. Changes again varied by site: two primary care clinics had a non-significant decrease in guideline concordance, and one had a significant increase; two emergency departments had significant increases in guideline concordance while one had no significant change. When stratified by diagnosis, a significant overall increase in guideline-concordant prescriptions was found for AOM, streptococcal pharyngitis, and UTI, though individual sites had variable results. The percent of guideline-concordant prescriptions for sinusitis and CAP did not differ between the study periods (Figure 2).

For all diagnoses combined, there was a significant increase in 5-day prescriptions (6.3% baseline vs 19.7% intervention, *P* < .001) and a significant decrease in 10-day prescriptions (67.7% baseline vs 54.3% intervention, *P* < .001) (Figure 2).

There was a significant decrease in rapid streptococcal pharyngitis testing (10.9% baseline vs 7.6% intervention, *P* < .001), a trend which was present in all sites aside from one primary care clinic.

The rate of return visits within 14 days was low and did not differ significantly between the periods (5849/43157 [13.6%] baseline vs 13591/100200 [13.6%] postimplementation; *P* = .96).

## Discussion

In this large prepost intervention study, we demonstrated that implementation of a bundled tele-stewardship intervention across rural and suburban pediatric primary care clinics and emergency departments was associated with lower antibiotic prescription rate and less diagnostic testing for streptococcal pharyngitis with mixed overall results for guideline-concordant prescribing. Our findings support the feasibility of tele-stewardship interventions in pediatric ambulatory rural settings, as has been previously reported for adults.^17^

The impact of the intervention was not similar across all practices. Outcomes including overall antibiotic prescription rate and guideline-concordant prescribing varied by site. The sites where the intervention did not reduce antibiotic prescribing did not significantly differ from others in provider or practice characteristics like number of clinicians, number of patients seen, or number of advanced practice providers. Data on how long each prescriber was in practice was not available but has been shown to impact antibiotic prescribing.^18^ We did not see associations between intervention effectiveness and ED or primary care location. Site C had more patients seen by advanced practice providers compared to the other primary care clinics and had a larger decrease in prescribing. Provider engagement in the intervention, as assessed by measuring utilization of educational modules also did not differ between sites and was low overall. We did not track site engagement with quarterly educational sessions but did coordinate the sessions in previously scheduled site-specific administration meetings and offer CME to increase attendance. However, sites were different in terms of patient demographics such as race, ethnicity, and primary spoken language. For example, the patient population at sites C and E (which both had decreases in overall antibiotic prescribing) was over 80% White, while the population of site B (which had an increase in overall antibiotic prescribing) was less than 40% White. Whether unconscious clinician bias, systemic racism, language barriers, patient expectations, and clinicians’ expectations of patients contributed to the variable outcomes at different sites requires further study.

While our study had many strengths including a large sample size, inclusion of multiple sites and outpatient settings, rural and suburban settings, analysis of multiple diagnoses, and a two-year intervention period to reduce the effects of seasonal variation in prescription rates, it also had several limitations. Approximately 10% of the prescriptions had missing information in the duration field and were excluded from the analysis. In the latter half of 2024, national and local rates of *Mycoplasma pneumoniae* increased far above their usual rates.^19^ Since this infection is usually treated with a 5-day course of azithromycin which is not considered to be a first-line antibiotic for CAP, high community prevalence of *Mycoplasma* may have lowered the rate of guideline concordance we found for CAP. However, we performed a sensitivity analysis of guideline concordance excluding the diagnosis of CAP and found similar results to the primary analysis. Another limitation was the delay in the roll-out of some of the interventions. Implementation of the Firstline application and dissemination of patient educational materials occurred in the latter half of the postimplementation period due to delays receiving institutional approvals, possibly limiting the effectiveness of the intervention. The intervention was bundled, so it is impossible to discern if any one of the components of the intervention was more effective than others. Finally, each patient’s diagnosis was dependent on the clinical judgement of their individual clinician, which could not practically be verified or standardized given the number of encounters involved.

Despite these limitations, the results of this study support the use of implementing bundled interventions using tele-stewardship to improve appropriate antimicrobial prescribing in pediatric rural and suburban primary care clinics and EDs. Further research into what factors drive the effectiveness of stewardship interventions in these settings would help sites tailor which parts of the bundle to implement for their needs. Tele-stewardship has great potential to improve appropriate antibiotic prescribing and quality of care in rural areas that lack access to in-person stewardship expertise.

## Supporting information

10.1017/ash.2026.10353.sm001 Peworchik et al. supplementary material Peworchik et al. supplementary material

## Supplementary material

The supplementary material for this article can be found at https://doi.org/10.1017/ash.2026.10353.

## References

[1] Antimicrobial Resistance Stats and Facts. Centers for Disease Control and Prevention. 2021. https://www.cdc.gov/antimicrobial-resistance/data-research/facts-stats/?CDC_AAref_Val= https://www.cdc.gov/drugresistance/national-estimates.html. Accessed December 18, 2024.

[2] Suda KJ , Hicks LA , Roberts RM , Hunkler RJ , Danziger LH. A national evaluation of antibiotic expenditures by healthcare setting in the United States, 2009. J Antimicrob Chemother 2013;68:715–718. 10.1093/jac/dks445. 23148204

[3] Dantuluri KL , Bruce J , Edwards KM , et al. Rurality of residence and inappropriate antibiotic use for acute respiratory infections among young Tennessee children. Open Forum Infect Dis 2021;8:ofaa587. 10.1093/ofid/ofaa587. 33511228 PMC7814393

[4] Mailig M , Cookson NA , Schulz LT. Telestewardship programs support clinical care and improve fiscal outcomes across the continuum through partnership between hospitals and health systems: a systematic review. Am J Health Syst Pharm 2022;79:1663–1673. 35773093 10.1093/ajhp/zxac179

[5] Pierce J , Stevens MP. The emerging role of telehealth in antimicrobial stewardship: a systematic review and perspective. Curr Treat Options Infect Dis 2021;13:175–191. 34975344 10.1007/s40506-021-00256-7 PMC8713008

[6] Gerber JS , Prasad PA , Fiks AG , et al. Effect of an outpatient antimicrobial stewardship intervention on broad-spectrum antibiotic prescribing by primary care pediatricians: a randomized trial. JAMA 2013;309:2345–2353. 23757082 10.1001/jama.2013.6287

[7] Oertli C , Staub M , Zhang M , Katz SE. Impact of mandatory indications for outpatient antibiotic orders on accurate tracking of antibiotic indications. Infect Control Hosp Epidemiol 2024;45:1–6. 10.1017/ice.2024.88 PMC11518663 38738537

[8] Lieberthal AS , Carroll AE , Chonmaitree T , et al. The diagnosis and management of acute otitis media. Pediatrics 2013;131:e964–e999. 23439909 10.1542/peds.2012-3488

[9] Shulman ST , Bisno AL , Clegg HW , et al. Clinical practice guideline for the diagnosis and management of group a streptococcal pharyngitis: 2012 update by the Infectious Diseases Society of America. Clin Infect Dis 2012;55:e86–102. 22965026 10.1093/cid/cis629 PMC7108032

[10] Bradley JS , Byington CL , Shah SS , et al. The management of community-acquired pneumonia in infants and children older than 3 months of age: clinical practice guidelines by the Pediatric Infectious Diseases Society and the Infectious Diseases Society of America. Clin Infect Dis 2011;53:e25–76. 21880587 10.1093/cid/cir531 PMC7107838

[11] Wald ER , Applegate KE , Bordley C , et al. Clinical practice guideline for the diagnosis and management of acute bacterial sinusitis in children aged 1 to 18 years. Pediatrics 2013;132:e262–e280. 23796742 10.1542/peds.2013-1071

[12] Dialogue Around Respiratory Illness Treatment (DART) – iMTR. https://www.uwimtr.org/dart/. Accessed May 12, 2021.

[13] Talking with Vaccine Hesitant Patients. AAP. https://www.aap.org/en/patient-care/immunizations/.

[14] QuizTime | Center for Advanced Mobile Healthcare Learning. https://www.vumc.org/camhl/quiztime. Accessed September 14, 2022.

[15] Firstline. Firstline - Clinical Decision Support Platform. https://firstline.org/. Accessed September 14, 2022.

[16] French B , Heagerty PJ. Analysis of longitudinal data to evaluate a policy change. Stat Med 2008;27:5005–5025. 18618416 10.1002/sim.3340 PMC3415557

[17] Stenehjem E , Wallin A , Willis P , et al. Implementation of an antibiotic stewardship initiative in a large urgent care network. JAMA Netw Open 2023;6:e2313011. 37166794 10.1001/jamanetworkopen.2023.13011 PMC10176123

[18] Baillie EJ , Merlo G , Van Driel ML , Magin PJ , Hall L. Early-career general practitioners’ antibiotic prescribing for acute infections: a systematic review. J Antimicrob Chemother 2024;79:512–525. 38252922 10.1093/jac/dkae002 PMC10904722

[19] Raghuram A , Furmanek S , Chandler T , Rashid S , Mattingly W , Ramirez J. Description of a current outbreak of *Mycoplasma pneumoniae* in the United States. Pathogens 2025;14:60. 39861021 10.3390/pathogens14010060 PMC11768315

