# Supplementary material for: The impact of tele-stewardship on rural and suburban pediatric ambulatory antibiotic prescribing
Source: Antimicrob Steward Healthc Epidemiol. 2026 Apr 21;6(1):e119. doi: 10.1017/ash.2026.10353 (PMC13104536; doi:10.1017/ash.2026.10353)

eTable. Percent change in overall antimicrobial prescribing by demographic

Table shows percent change in antibiotic prescription rates from baseline period to implementation period at all sites broken down by race, ethnicity, primary spoken language, and insurance status. Groups that had a significant improvement in their prescription rate are marked with an asterisk.

|  | Baseline | Implementation | % Change | p-value |
| --- | --- | --- | --- | --- |
| **Race** |  |  |  |  |
| Black | 9.6% | 11.1% | +1.5% | <0.001 |
| White | 13.6% | 12.2% | -1.5% | <0.001* |
| Other | 10.5% | 12% | +1.5% | 0.002 |
| Unknown | 10.6% | 10.5% | -0.2% | 0.829 |
| **Ethnicity** |  |  |  |  |
| Hispanic/Latino | 12% | 12.3% | +0.2% | 0.63 |
| Non-Hispanic/Latino | 12.5% | 11.9% | -0.6% | 0.01* |
| Unknown | 12.2% | 11.3% | -1.0% | 0.034* |
| **Primary Language** |  |  |  |  |
| English | 12.7% | 11.8% | -0.9% | <0.001* |
| Non-English | 10.6% | 12.4% | +1.8% | <0.001 |
| Unknown | 6.1% | 6.7% | +0.6% | 0.909 |
| **Insurance Status** |  |  |  |  |
| Private | 12.8% | 12% | -0.8% | 0.039* |
| Public | 13.1% | 12.6% | -0.5% | 0.021* |
| Self-Pay | 17.6% | 19.7% | +2% | 0.212 |
| Unknown | 6.1% | 5.3% | +0.1% | 0.886 |

*denotes statistically significant improvement

eFigure: Example of Individualized Feedback. Example of email sent to providers quarterly.


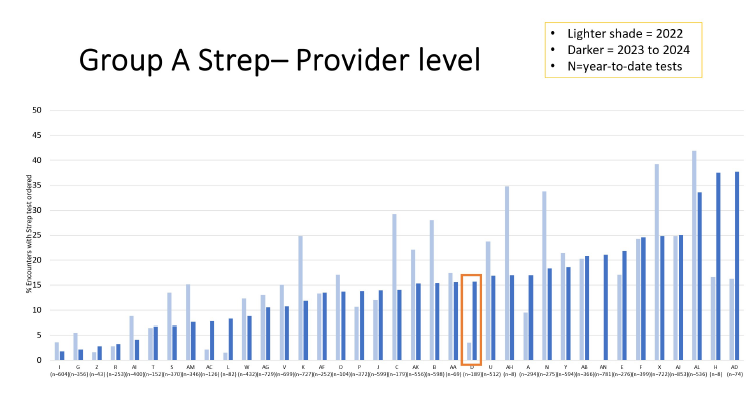

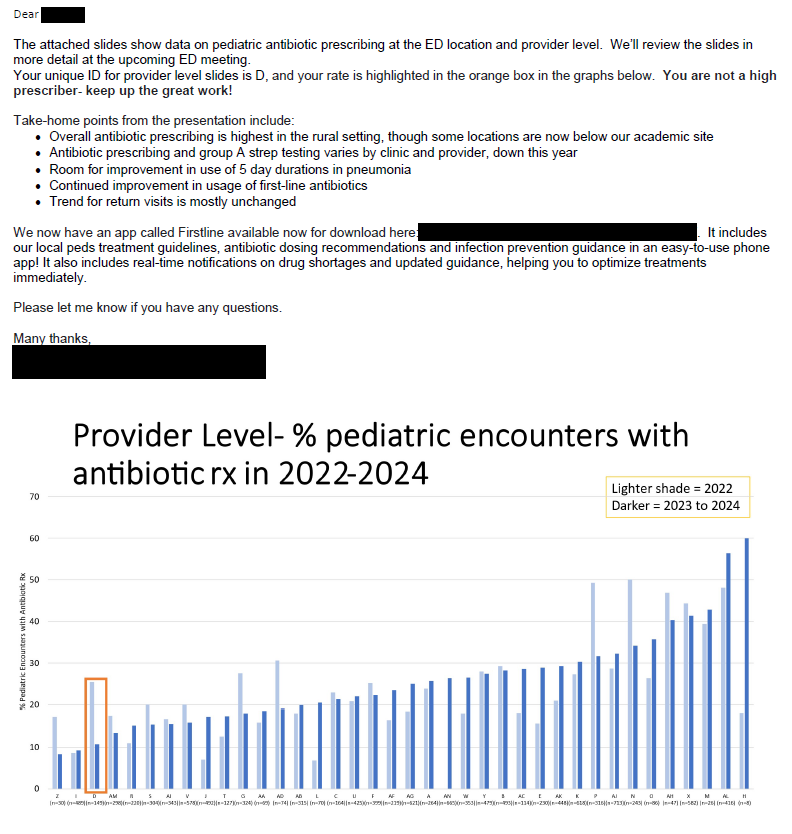

Supplement: Peworchik et al. supplementary material [file S2732494X26103532sup001.docx]
